# Knowledge, attitudes and practices towards cardiac rehabilitation among patients with heart failure

**DOI:** 10.1186/s12889-026-27912-1

**Published:** 2026-05-29

**Authors:** Fang Wei, Runze Huang, Wei Li

**Affiliations:** Department of cardiovascular medicine, Affiliated Hospital of Guizhou Medical University, Guiyang, 550001 China

**Keywords:** Heart failure, Cardiac rehabilitation, Knowledge, Attitudes, Practice, Cross-sectional study, Patient education

## Abstract

**Background:**

This study aimed to examine the knowledge, attitudes, and practices (KAP) related to cardiac rehabilitation (CR) among patients with heart failure (HF).

**Methods:**

A cross-sectional study was conducted among 589 patients with HF at the Affiliated Hospital of Guizhou Medical University from August 31, 2025 to November 18, 2025. Demographic information and KAP scores were obtained using a self-designed questionnaire. Structural equation modeling (SEM) was applied to assess the associations among the KAP components.

**Results:**

A total of 589 valid questionnaires were included in the analysis, yielding an effective response rate of 88.57%. Of the participants, 306 (51.95%) were male, and the mean age was 49.89 ± 14.30 years. The mean scores for knowledge, attitude, and practice were 6.48 ± 2.50 (range: 0–11), 29.36 ± 4.50 (range: 8–40), and 28.18 ± 7.35 (range: 8–40), respectively. SEM analysis indicated that knowledge had a direct positive effect on attitude (β = 0.87, *P* < 0.001) and a direct negative effect on practice (β = −1.06, *P* < 0.001), whereas attitude showed a direct positive effect on practice (β = 0.78, *P* < 0.001). Moreover, knowledge exerted a positive indirect effect on practice through attitude (β = 0.68, *P* = 0.001).

**Conclusion:**

Patients with HF demonstrated moderate knowledge levels, generally positive attitudes, and relatively active practices regarding cardiac rehabilitation. The findings indicate that the association between knowledge and practice is not linear, with attitudes serving as an important mediating factor. These results suggest that efforts to enhance participation in cardiac rehabilitation should focus not only on improving patient knowledge but also on addressing attitudinal and contextual influences that affect the translation of knowledge into practice.

**Supplementary Information:**

The online version contains supplementary material available at https://doi.org/10.1186/s12889-026-27912-1.

## Background

Heart failure (HF) is a complex clinical syndrome resulting from impaired cardiac pump function and remains a major global public health challenge. Reduced cardiac output limits the heart’s capacity to meet the metabolic demands of the body, leading to a spectrum of typical symptoms and clinical signs [1]. For many years, patients with HF were advised to avoid physical activity because of concerns about safety and the potential worsening of symptoms [1]. However, this traditional view has been increasingly questioned by evidence from randomized controlled trials. Notably, the HF-ACTION trial confirmed that structured exercise training is safe for patients with HF and does not increase the risk of adverse clinical events [2]. In addition, subsequent studies have shown that combined endurance and resistance training can significantly improve exercise tolerance and muscle strength [3], with subgroup analyses indicating that these benefits are observed in both men and women [4].

Strong evidence supporting exercise-based cardiac rehabilitation (CR) was further reinforced by a comprehensive Cochrane systematic review published in 2023 [5]. This meta-analysis, which included 60 randomized controlled trials, demonstrated that participation in CR was associated with a 31% reduction in the risk of all-cause hospitalization. The clinical importance of this effect is underscored by a number needed to treat of 13, meaning that one hospitalization could be prevented for every 13 patients undergoing CR. Moreover, CR was associated with clinically meaningful improvements in quality of life, as indicated by a 7.39-point increase in the Minnesota Living with Heart Failure Questionnaire score, exceeding the established minimal clinically important difference of 5 points.

Despite these well-established benefits, participation in CR remains suboptimal worldwide. In the United States and Europe, less than 20% of eligible patients are reported to take part in rehabilitation programs [6–8]. Multiple factors have been implicated in this low uptake, including constraints within healthcare systems, insufficient awareness among clinicians, and barriers at the patient level [9]. In highly populous countries such as China, limited availability of trained CR professionals has been identified as an additional obstacle to sustained participation [10].

The Knowledge, Attitude, and Practice (KAP) model offers a useful theoretical framework for exploring health-related behaviors [11]. According to this model, health practices are shaped by both an individual’s level of knowledge and their underlying attitudes. Although the effectiveness of CR in HF management is well supported, persistently low participation rates highlight the importance of gaining a deeper understanding of patient-related barriers. Systematic evaluation of knowledge, attitudes, and practices may help identify key targets for intervention and inform strategies aimed at improving adherence and clinical outcomes.

By focusing on the interrelationships among knowledge, attitudes, and practices, this approach may also provide broader insights into patient-level challenges and contribute to the optimization of CR programs across different healthcare settings. Accordingly, the present study aimed to assess the knowledge, attitudes, and practices of patients with heart failure regarding cardiac rehabilitation.

## Methods

### Study design and participants

This cross-sectional study was carried out among patients with HF at the Affiliated Hospital of Guizhou Medical University between August 31, 2025 and November 18, 2025. Ethical approval was obtained from the Ethics Committee of the Affiliated Hospital of Guizhou Medical University (2025122 K), and informed consent was secured from all participants before they completed the survey. The inclusion criteria were: (1) a confirmed diagnosis of HF in accordance with the Chinese Guidelines for the Diagnosis and Treatment of Heart Failure 2024 [12]; (2) age over 18 years and voluntary agreement to participate; and (3) sufficient communication skills to understand the study and respond accurately. Exclusion criteria included: (1) diagnosis of a mental disorder; and (2) presence of severe systemic diseases or complications that might interfere with treatment outcomes or survey responses.

### Questionnaire

The questionnaire was a self-designed instrument developed for this study based on the “2022 AHA/ACC/HFSA Guideline for the Management of Heart Failure: A Report of the American College of Cardiology/American Heart Association Joint Committee on Clinical Practice Guidelines“ [13] and was not adopted from a previously validated scale. A preliminary survey of 30 participants was conducted to test the draft, which demonstrated good internal consistency, with an overall Cronbach’s α of 0.883, supporting the reliability of the instrument. Content validity was ensured by developing the questionnaire based on established clinical guidelines and by assessing the clarity, comprehensibility, and relevance of the items during the pilot testing phase. In the formal investigation, all eligible participants received a face-to-face explanation of the study purpose and questionnaire requirements from trained researchers before completing the survey, in order to improve the consistency and appropriateness of questionnaire completion.

The final questionnaire was administered in Chinese and comprised four sections: demographic information (including gender, age, height, weight, education, income, and medical history), and assessments of knowledge, attitudes, and practices. The knowledge section contained 11 items, scored as 1 for a correct answer and 0 for an incorrect or unclear response, resulting in a total possible score of 0–11. In this study, “knowledge” refers to patients’ understanding of heart failure and cardiac rehabilitation, including awareness of symptoms, treatment principles, and the role of rehabilitation in disease management. The attitude section included 8 items rated on a five-point Likert scale from “strongly agree” to “strongly disagree,” scored 5 to 1, with a total possible score ranging from 8 to 40. In this study, “attitudes” refer to patients’ beliefs, perceptions, and willingness toward cardiac rehabilitation, including their recognition of its importance and their confidence in engaging with CR-related recommendations. The practice section also contained 8 items, scored from 5 (“always”) to 1 (“never”), yielding a score range of 8–40. “Practices” refer to patients’ actual engagement in CR-related behaviors, such as adherence to prescribed medication, maintenance of a healthy diet, regular symptom monitoring, and participation in recommended rehabilitation activities. Scores exceeding 70% of the maximum possible in each section were considered indicative of sufficient knowledge, positive attitudes, and proactive practices, based on commonly used criteria in KAP studies [14].

### Questionnaire distribution and quality control

The questionnaire was primarily distributed through online channels, targeting patients at the study center. Specifically, participants completed the survey electronically on their mobile phones by scanning a QR code generated via the Sojump platform (https://www.wjx.cn/). The research team communicated the study objectives to the heads of hospital departments, obtaining their consent and cooperation. A convenience sampling method was employed, and the research team conducted face-to-face interactions with all admitted patients. Each participant received a detailed explanation of the study’s aims and procedures. Once eligibility was confirmed and consent obtained, a QR code was provided to access the electronic questionnaire. To prevent multiple submissions, an IP address restriction was applied, allowing each survey to be completed only once per IP address. After data collection, the research team performed a quality check of the questionnaires. Responses completed in less than 90 s, those with evident logical inconsistencies, or those showing repetitive answer patterns were considered invalid and excluded from the analysis. These measures were implemented to ensure the authenticity and reliability of the collected data. Together, these procedures were used to improve the suitability of the questionnaire for patients with HF and to enhance the quality of the final dataset.

### Sample size

The required sample size was estimated using the formula for cross-sectional studies [15]: α = 0.05, $\:\mathrm{n=}{\left(\frac{{\mathrm{Z}}_{\mathrm{1-}\alpha{/2}}}{\delta}\right)}^{\mathrm{2}}\times{p}\times\left(\mathrm{1-}\mathrm{p}\right)$ where $\:{\mathrm{Z}}_{\mathrm{1-}\alpha{/2}}$=1.96 for α = 0.05, the assumed degree of variability of *p*=0.5 maximizes the required sample size, and δ is admissible error (which was 5% here). Based on this calculation, the theoretical sample size was 480. An additional 20% was added to account for potential dropouts or incomplete responses, ensuring adequate statistical power.

### Statistical analysis

All statistical analyses were conducted using Stata 17.0 (Stata Corporation, College Station, TX, USA). The normality of continuous variables was assessed using the Kolmogorov–Smirnov test. Variables following a normal distribution were expressed as mean ± standard deviation (SD) and compared using the Student’s t-test for two-group comparisons or one-way analysis of variance (ANOVA) for comparisons involving more than two groups. Categorical variables were presented as counts and percentages (n [%]). Spearman correlation analysis was applied to evaluate the relationships among knowledge (K), attitude (A), and practice (P). Correlation strength was interpreted as follows: weak = 0.10–0.39, moderate = 0.40–0.69, strong = 0.70–0.89, and very strong = 0.90–1.00.

Variables with P-values < 0.05 in univariate analyses were subsequently included in multivariate regression analyses. The median value of the practice score was used as the cutoff for dichotomization. Structural equation modeling (SEM) was performed based on the following hypotheses: (1) knowledge influences both attitude and practice; and (2) attitude influences practice. P-values were reported to three decimal places, and a two-sided P-value < 0.05 was considered statistically significant.

## Results

### Demographic characteristics

A total of 665 questionnaires were initially collected. After screening, several responses were excluded: 3 participants declined to take part, 11 questionnaires were completed in under 90 s, 2 were submitted by individuals of atypical age, and 59 contained illogical responses. Ultimately, 589 valid questionnaires were retained for analysis, corresponding to an effective response rate of 88.57%. Among the participants, the mean age was 49.89 ± 14.30 years, with 306 (51.95%) male. The average BMI was 22.92 ± 3.08 kg/m². In terms of sociodemographic characteristics, 322 participants (54.67%) lived in urban areas, 298 (50.59%) had an associate degree or higher, and 246 (41.77%) reported a household per capita monthly income of 5,000–10,000 CNY. Regarding lifestyle and health conditions, 243 participants (41.26%) were smokers, 232 (39.39%) consumed alcohol, 216 (36.67%) did not maintain regular exercise habits, 266 (45.16%) had hypertension, and 264 (44.82%) had coronary heart disease (Table 1).

**Table 1:** Demographic characteristics and knowledge, attitude, and practice scores

	*N* (%)	Knowledge score		Attitude score		Practice score	
		Mean ± SD	*P*	Mean ± SD	*P*	Mean ± SD	*P*
Total score		6.48 ± 2.50		29.36 ± 4.50		28.18 ± 7.35	
Gender			0.416		0.288		0.059
Male	306 (51.95)	6.38 ± 2.53		29.18 ± 4.36		28.73 ± 7.17	
Female	283 (48.05)	6.57 ± 2.44		29.55 ± 4.64		27.58 ± 7.48	
Age (years old) [range: 20 ~ 89]	49.89 ± 14.30						
Residence			**0.021**		**0.0002**		**0.012**
Rural	267 (45.33)	6.20 ± 2.56		28.61 ± 4.62		29.03 ± 7.28	
Urban	322 (54.67)	6.70 ± 2.41		29.98 ± 4.31		27.47 ± 7.33	
Marital status			0.329		0.456		0.605
Married	431 (73.17)	6.40 ± 2.51		29.43 ± 4.56		28.29 ± 7.26	
Other (Divorced or Widowed)	158 (26.83)	6.67 ± 2.44		29.15 ± 4.33		27.88 ± 7.57	
Education			**0.010**		**0.019**		0.067
Middle school or below	139 (23.6)	5.97 ± 2.68		29.10 ± 4.08		27.56 ± 7.51	
High school or technical school	152 (25.81)	6.30 ± 2.21		28.76 ± 4.69		29.35 ± 7.25	
Associate/bachelor’s degree or above	298 (50.59)	6.79 ± 2.50		29.78 ± 4.55		27.87 ± 7.26	
Type of medical insurance (multiple choices allowed):							
No insurance	73 (12.39)						
Only social insurance	308 (52.29)						
Social insurance & other commercial insurance	306 (51.95)						
Monthly income			0.863		0.592		**0.003**
< 2000	56 (9.51)	5.91 ± 3.34		29.14 ± 4.89		26.58 ± 6.65	
2000–5000	182 (30.9)	6.42 ± 2.69		29.48 ± 4.17		26.88 ± 7.83	
5000–10,000	246 (41.77)	6.55 ± 2.20		29.21 ± 4.56		29.36 ± 6.99	
> 10,000	105 (17.83)	6.68 ± 2.23		29.62 ± 4.72		28.50 ± 7.18	
Smoking			**0.002**		**0.003**		**0.008**
Yes	243 (41.26)	6.15 ± 2.37		28.75 ± 4.44		29.14 ± 7.24	
No	346 (58.74)	6.70 ± 2.55		29.78 ± 4.50		27.50 ± 7.35	
Do you drink alcohol?			0.489		0.547		0.314
Yes	232 (39.39)	6.46 ± 2.41		29.23 ± 4.43		28.48 ± 7.32	
No	357 (60.61)	6.48 ± 2.54		29.44 ± 4.55		27.98 ± 7.36	
Do you have a regular exercise habit?			0.621		0.072		0.240
Yes	373 (63.33)	6.49 ± 2.35		29.06 ± 4.60		28.45 ± 7.12	
No	216 (36.67)	6.44 ± 2.72		29.87 ± 4.28		27.71 ± 7.70	
BMI	22.92 ± 3.08						
Do you have any of the following conditions? (multiple choices allowed)							
Diabetes	177 (30.05)						
Coronary heart disease	264 (44.82)						
Hyperlipidemia	190 (32.26)						
Hypertension	266 (45.16)						
Other	98 (16.64)						
None of the above	135 (22.92)						

Bold values indicate statistically significant differences, *P* < 0.05

### Knowledge, attitude, and practice dimensions

The mean scores for knowledge, attitude, and practice were 6.48 ± 2.50 (range: 0–11), 29.36 ± 4.50 (range: 8–40), and 28.18 ± 7.35 (range: 8–40), respectively. Knowledge scores varied significantly by residence type (*P* = 0.021), education level (*P* = 0.010), and smoking status (*P* = 0.002). Attitude scores showed significant differences according to residence type (*P* = 0.0002), education (*P* = 0.019), and smoking status (*P* = 0.003). Practice scores differed significantly based on residence type (*P* = 0.012), household per capita monthly income (*P* = 0.003), and smoking status (*P* = 0.008) (Table 1).

Within the knowledge dimension, the item with the highest error rate was “The symptoms of heart failure are markedly evident” (K4) at 59.42%, whereas the question with the largest proportion of “Unclear” responses was “HF is the severe and terminal stage of all cardiovascular diseases” (K1) at 30.73% (Supplementary Table S1). For attitudes, 21.56% strongly agreed and 21.05% agreed that CR is mainly managed by doctors and patients generally do not need to pay much attention (A2). Additionally, 21.22% strongly agreed and 22.58% agreed that CR requires considerable effort and they lacked confidence in strictly following it (A3) (Supplementary Table S2). Regarding practices, 16.98% of participants reported never adhering to prescribed medication schedules and dosages (P8), 16.64% never maintained a healthy diet (P4), and 15.62% never monitored themselves for potential HF symptoms (P2) (Supplementary Table S3).

### Spearman correlation analysis

Spearman correlation analysis revealed significant positive associations between knowledge and attitude (*r* = 0.378, *P* < 0.001) and between attitude and practice (*r* = 0.103, *P* = 0.013). No significant correlation was observed between knowledge and practice (*r* = -0.053, *P* = 0.190) (Table 2).

**Table 2:** Spearman correlation analysis

	Knowledge	Attitude	Practice
Knowledge	1		
Attitude	0.3782 (*P*<0.001)	1	
Practice	-0.0530 (*P* = 0.190)	0.1027 (*P* = 0.013)	1

### Univariable and multivariable logistic regression analysis

Multivariable logistic regression analysis indicated that several factors were independently associated with proactive practice. These included knowledge scores (OR = 0.85, 95% CI: 0.79–0.92, *P* < 0.001), attitude scores (OR = 1.14, 95% CI: 1.09–1.19, *P* < 0.001), urban residence (OR = 0.50, 95% CI: 0.34–0.74, *P* < 0.001), education level of high school/technical secondary school compared with junior high school or below (OR = 2.02, 95% CI: 1.20–3.41, *P* = 0.008), household per capita monthly income of 5,000–10,000 CNY compared with < 2,000 CNY (OR = 4.34, 95% CI: 2.16–8.69, *P* < 0.001), household per capita monthly income > 10,000 CNY compared with < 2,000 CNY (OR = 2.85, 95% CI: 1.34–6.06, *P* = 0.006), smoking status (OR = 1.79, 95% CI: 1.23–2.63, *P* = 0.002), and BMI (OR = 0.86, 95% CI: 0.81–0.92, *P* < 0.001) (Table 3).

**Table 3:** Univariable and multivariable logistic regression analysis

Practice	Univariate analysis		Multivariate analysis	
	OR (95%CI)	*P*	OR (95%CI)	*P*
Knowledge score	0.92 (0.86, 0.98)	0.015	0.85 (0.79, 0.92)	< 0.001
Attitude score	1.06 (1.02, 1.10)	0.001	1.14 (1.09, 1.19)	< 0.001
Gender				
Male				
Female	0.84 (0.60, 1.16)	0.296		
Age(years old)[range: 20 ~ 89]	1.00 (0.99, 1.01)	0.364		
Residence				
Rural				
Urban	0.65 (0.47, 0.90)	0.01	0.50 (0.34, 0.74)	< 0.001
Marital status				
Married				
Other (Divorced or Widowed)	0.97 (0.67, 1.40)	0.883		
Education				
Middle school or below				
High school or technical school	2.02 (1.27, 3.23)	0.003	2.02 (1.20, 3.41)	0.008
Associate/bachelor’s degree or above	1.27 (0.84, 1.90)	0.247	1.16 (0.73, 1.85)	0.518
Monthly income				
< 2000				
2000–5000	1.46 (0.77, 2.79)	0.242	1.54 (0.77, 3.08)	0.219
5000–10,000	3.52 (1.88, 6.57)	< 0.001	4.34 (2.16, 8.69)	< 0.001
> 10,000	2.33 (1.17, 4.64)	0.015	2.85 (1.34, 6.06)	0.006
Smoking				
Yes	1.79 (1.28, 2.5)	0.001	1.79 (1.23, 2.63)	0.002
No				
Do you drink alcohol?				
Yes	1.30 (0.93, 1.82)	0.139		
No				
Do you have a regular exercise habit?				
Yes	1.20 (0.86, 1.69)	0.278		
No				
BMI	0.89 (0.84, 0.94)	< 0.001	0.86 (0.81, 0.92)	< 0.001

### SEM analysis

**Fig. 1:**
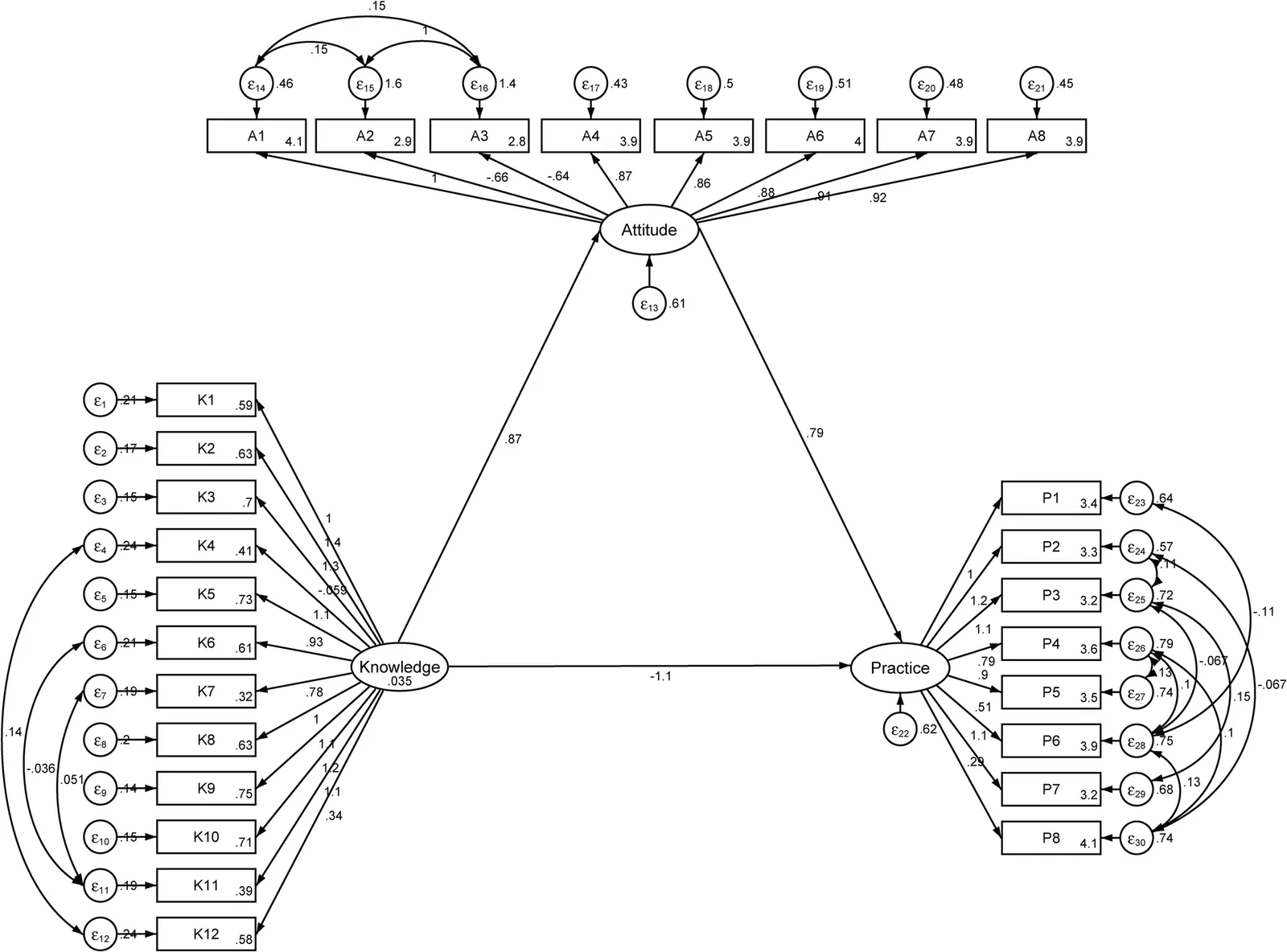
Graphical representation of the structural equation model

Structural equation modeling (SEM) demonstrated acceptable model fit. The model fit indices were as follows: RMSEA = 0.060 (reference < 0.08, good), SRMR = 0.093 (reference < 0.08, good), TLI = 0.870 (reference > 0.8, good), and CFI = 0.886 (reference > 0.8, good), suggesting that the proposed model fit the data reasonably well (Supplementary Table S4). SEM results indicated that knowledge had a direct positive effect on attitude (β = 0.870, *P* < 0.001), attitude directly influenced practice (β = 0.780, *P* < 0.001), and knowledge exerted a direct negative effect on practice (β = -1.060, *P* < 0.001). In addition, knowledge indirectly affected practice through attitude (β = 0.680, *P* = 0.001) (Table 4; Fig. 1).

**Table 4:** SEM analysis

Model paths		Total effects		Direct Effect		Indirect effect	
		β (95% CI)	*P*	β (95% CI)	*P*	β (95% CI)	*P*
Asum <-							
	Ksum	0.87(0.39,1.34)	< 0.001	0.87(0.39,1.34)	< 0.001		
Psum <-							
	Asum	0.78(0.66,0.91)	< 0.001	0.78(0.66,0.91)	< 0.001		
	Ksum	-0.37(-0.93,0.18)	0.192	-1.06(-1.61,-0.50)	< 0.001	0.68(0.29,1.07)	0.001

## Discussion

Our findings suggest that patients with HF demonstrate moderate levels of knowledge, accompanied by generally positive attitudes and proactive behaviors toward CR. However, the observed relationships between knowledge and practice should be interpreted cautiously, as the cross-sectional design precludes causal inference, and these associations may be influenced by various contextual factors.

This study highlights key challenges in the KAP of HF patients regarding CR, identifying specific areas that warrant targeted interventions. A notable knowledge deficit was observed, particularly concerning nuanced aspects of CR, including the role of exercise rehabilitation and the importance of self-management. Although attitudes and practices were generally favorable, the knowledge gap indicates that many patients may not fully understand the rationale behind rehabilitation strategies, potentially hindering adherence to recommended behaviors. Similar challenges have been reported in other healthcare contexts, where limited health literacy has been identified as a barrier to effective disease management and long-term adherence to CR programs [16, 17]. Compared with recent studies, our results show some notable differences. Gao et al. found that among patients with chronic heart failure and their caregivers, knowledge, attitude, and practice regarding CR were all positively interrelated, with knowledge exerting both direct and indirect positive effects on practice in their SEM analysis [18]. Another cross-sectional study of patients with chronic heart failure focusing on home-based CR also reported positive correlations among KAP dimensions, with knowledge influencing practice both directly and indirectly through attitudes [19]. In contrast, the present study observed a negative direct effect of knowledge on practice and a non-significant total effect in SEM analysis. These discrepancies may reflect differences in study populations, settings, questionnaire design, and cultural or healthcare system contexts that shape patient engagement. Collectively, these findings underscore the need for educational interventions that provide patients with the knowledge required to actively participate in their care. In addition, evidence from studies conducted in settings with higher CR adherence rates suggests that structured, multidisciplinary programs and coordinated referral systems are key contributors to improved patient engagement [20]. For example, populations enrolled in comprehensive CR models with integrated exercise training, patient education, and follow-up support have demonstrated higher participation and adherence levels [21]. These findings, interpreted within the KAP framework, indicate that beyond knowledge acquisition, sustained engagement is closely linked to supportive healthcare structures and reinforcement mechanisms that strengthen both attitudes and behavioral implementation [22]. Compared with such settings, the relatively lower and less consistent engagement observed in the present study may reflect gaps in program accessibility, continuity of care, and behavioral support, highlighting potential areas for targeted improvement.

The interplay between knowledge, attitudes, and practices demonstrates a complex dynamic in CR participation. The lack of a significant direct association between knowledge and practice in Spearman correlation analysis aligns with the non-significant total effect of knowledge on practice in the SEM model, further emphasizing the complexity of their relationship. SEM results indicated that while knowledge had a strong positive effect on attitudes, its direct influence on practice was negative, whereas the indirect effect via attitudes was positive. The total effect of knowledge on practice was not statistically significant, suggesting that these opposing pathways may counterbalance each other, and that knowledge alone does not exert a consistent overall influence on practice in this cross-sectional setting [23, 24].

Several factors may explain this seemingly counterintuitive finding. First, certain knowledge items may not directly translate into actionable behaviors and might instead reflect heightened awareness of disease severity or perceived barriers, which could discourage engagement. Second, unmeasured confounding variables, such as functional limitations, disease severity, access to rehabilitation services, or constraints within the healthcare system, may simultaneously influence both knowledge and practice. Third, the different analytical approaches applied in this study capture distinct facets of the knowledge–practice relationship, emphasizing its complexity rather than suggesting a straightforward causal pathway.

Socio-demographic factors are essential for understanding patterns of engagement in CR. Interestingly, patients residing in rural areas showed higher odds of proactive engagement, despite often facing limited access to specialized healthcare services. This paradoxical observation may be attributable to unmeasured contextual factors, such as stronger community support, more regular daily routines, or greater reliance on self-management in rural settings, warranting further investigation. These findings suggest that contextual and structural determinants, including access to services and community support, may play a pivotal role in shaping engagement patterns, particularly in rural populations. The observed link between educational attainment and engagement practices may reflect disparities in healthcare resource access rather than differences in knowledge alone [25, 26]. This interpretation is consistent with our observation that practical engagement often precedes and reinforces knowledge acquisition, highlighting that improving access to CR activities may be more impactful than conventional educational interventions alone.

Income also emerged as a significant predictor of engagement behaviors, with higher household incomes associated with more proactive practices. This aligns with evidence from similar populations, indicating that financial stability reduces barriers to healthcare access and facilitates lifestyle modifications, such as adopting healthier diets and participating in exercise programs [27, 28]. Conversely, the observed association between smoking and lower engagement underscores the role of entrenched behavioral patterns in hindering rehabilitation efforts. Smoking cessation, a key component of CR, requires comprehensive interventions that address both physiological dependence and psychological factors [29, 30]. Furthermore, body mass index (BMI) was inversely related to proactive practices, suggesting that patients with higher BMI face both physical and psychological challenges in implementing recommended lifestyle changes. This finding is consistent with prior research indicating that obesity imposes functional limitations and contributes to stigma and reduced self-efficacy, complicating adherence to rehabilitation programs [31, 32].

When examining responses within specific KAP dimensions, several notable patterns emerge. A considerable proportion of patients reported the belief that CR should be managed solely by healthcare providers. This perception highlights a gap in promoting patient engagement and self-management in current educational efforts. It also reflects a broader trend in healthcare where paternalistic models prevail, especially in contexts that do not actively foster patient autonomy [33, 34]. In addition, the negative responses regarding the integration of psychological counseling into rehabilitation programs underscore the need for a holistic approach addressing both physical and emotional aspects of heart failure management. Although often overlooked, psychological counseling has been shown to improve patient outcomes by reducing anxiety, enhancing motivation, and supporting adherence to lifestyle modifications [35, 36].

These findings also point to systemic challenges within the healthcare framework that may shape the observed patterns. Limited resource availability, uneven distribution of healthcare infrastructure, and fragmented care delivery models contribute to the disparities identified in this study. Evidence from other settings suggests that healthcare systems featuring integrated care models and comprehensive patient education programs achieve higher levels of patient engagement and adherence [37, 38]. For example, countries with well-established CR programs often report greater participation rates and improved outcomes, driven by coordinated efforts among primary care providers, specialized rehabilitation centers, and community-based initiatives [39, 40].

Addressing these gaps requires a multi-faceted strategy that combines systemic reforms with targeted interventions. At the organizational level, healthcare institutions should prioritize integrated care models that facilitate communication between patients and providers, improve access to rehabilitation services, and promote patient-centered care. This may include expanding CR program availability in underserved regions and leveraging digital health tools to overcome geographic and logistical barriers. In parallel, community-based initiatives, such as peer support networks and local education campaigns, can be instrumental in enhancing patient engagement, particularly in rural and low-income settings [41, 42].

This approach should prioritize the reduction of practical and structural barriers to participation in CR activities, while embedding educational components within routine care. Rather than being delivered as standalone interventions, educational elements should be seamlessly integrated into patients’ everyday experiences, such as during exercise sessions, dietary workshops, and routine monitoring activities [41, 42].

This study has several notable limitations. First, the cross-sectional design precludes the establishment of causal relationships among knowledge, attitudes, and practices. Second, reliance on self-reported questionnaires may introduce response bias, potentially affecting the accuracy of the collected data. Third, the study was conducted at a single institution, which may limit the generalizability of the findings to populations with varying socioeconomic and cultural backgrounds. Future research should explore how practical engagement influences knowledge acquisition within CR programs, particularly examining the role of early screening and immediate involvement in shaping long-term outcomes. Longitudinal studies are warranted to investigate the temporal dynamics between practical participation and knowledge development, while also considering the influence of systematic screening programs on early engagement and sustained adherence.

## Conclusion

Patients with HF demonstrated moderate knowledge levels, along with generally positive attitudes and relatively proactive behaviors regarding cardiac rehabilitation. The findings indicate that the relationship between knowledge and practice is complex, with attitudes serving as a key mediator, underscoring the multifaceted nature of behavioral engagement in CR. These results suggest that enhancing CR participation may require not only improving patient knowledge but also addressing attitudinal and contextual factors that affect the translation of knowledge into practice. Overall, this study provides evidence supporting the implementation of more integrated, patient-centered approaches to CR that consider both individual perceptions and real-world barriers to sustained engagement.

## Supplementary information

Below is the link to the electronic supplementary material.

Supplementary Material 1.

Supplementary Material 2.

## Data Availability

All data generated or analysed during this study are included in this published article.

## Abbreviations

KAP: Knowledge, attitudes, and practices
CR: Cardiac rehabilitation
HF: Heart failure
SEM: Structural equation model
SD: Standard deviation
