# Supplementary material for: Knowledge, attitudes and practices towards cardiac rehabilitation among patients with heart failure
Source: BMC Public Health. 2026 May 29;26:2779. doi: 10.1186/s12889-026-27912-1 (PMC13637361; doi:10.1186/s12889-026-27912-1)
Supplement: Supplementary file 1 [file 12889_2026_27912_MOESM1_ESM.docx]

**Part 1 Demographic characteristics**

**Gender**

Male

Female

**Age (years old) [range: 20~89]**

**Residence**

Rural

Urban

**Marital status**

Married

Other (Divorced or Widowed)

**Education**

Middle school or below

High school or technical school

Associate/bachelor’s degree or above

**Type of medical insurance (multiple choices allowed):**

No insurance

Only social insurance

Social insurance & other commercial insurance

**Monthly income**

< 2000

2000 - 5000

5000 - 10000

>10000

**Smoking**

Yes

No

**Do you drink alcohol?**

Yes

No

**Do you have a regular exercise habit?**

Yes

No

**BMI**

**Do you have any of the following conditions? (multiple choices allowed)**

Diabetes

Coronary heart disease

Hyperlipidemia

Hypertension

Other

None of the above

**Part 2 Knowledge**

| **1.** **Heart failure is the severe and terminal stage of all cardiovascular diseases.** | **True** | **False** | **Unclear** |
| --- | --- | --- | --- |
| **2.** **High incidence, high hospitalization rate, and high mortality rate are the main Epidemiological features of** **heart failure.** | **True** | **False** | **Unclear** |
| **3.** **When experiencing symptoms like chest tightness, shortness of breath, and fatigue, one should be alert to the occurrence of heart failure.** | **True** | **False** | **Unclear** |
| **4.** **Symptoms in Patients with heart failure are always obvious.** | **True** | **False** | **Unclear** |
| **5.** **There are many aspects of cardiac rehabilitation, including exercise training, lifestyle improvement, and psychological counseling, etc.** | **True** | **False** | **Unclear** |
| **6.** **Exercise rehabilitation is an important method of cardiac rehabilitation for patients with heart failure.** | **True** | **False** | **Unclear** |
| **7.** **Patients with malignant arrhythmias or acute heart failure can still participate in exercise rehabilitation.** | **True** | **False** | **Unclear** |
| **8.** **Common types of exercise rehabilitation include aerobic training (e.g., jogging), resistance training (e.g., shoulder press), and flexibility training.** | **True** | **False** | **Unclear** |
| **9.** **Cardiac rehabilitation generally requires regular assessment and timely adjustments based on actual conditions.** | **True** | **False** | **Unclear** |
| **10.** **Cardiac rehabilitation in heart failure patients should pay close attention to the indications and adverse reactions of drug therapy.** | **True** | **False** | **Unclear** |
| **11.** **Cardiac rehabilitation for Patients with heart failure can be solely managed by hospitals and doctors, with no need for the patient to engage much in self-management.** | **True** | **False** | **Unclear** |

**Part 3 Attitudes**

| **1.** **You believe that it is important for patients to pay attention to cardiac rehabilitation and to learn relevant knowledge.** | Strongly agree | Agree | Neutral | Disagree | Strongly disagree |
| --- | --- | --- | --- | --- | --- |
| **2.** **You believe that cardiac rehabilitation is mainly handled by doctors, and patients generally do not need to pay much attention.** | Strongly agree | Agree | Neutral | Disagree | Strongly disagree |
| **3.** **You think that cardiac rehabilitation will require a lot of effort, and you lack confidence in strictly adhering to it.** | Strongly agree | Agree | Neutral | Disagree | Strongly disagree |
| **4.** **You are willing to engage in cardiac rehabilitation through appropriate physical activities (e.g., jogging, weight-lifting, or flexibility exercises).** | Strongly agree | Agree | Neutral | Disagree | Strongly disagree |
| **5.** **You pay significant attention to weight management in your daily life.** | Strongly agree | Agree | Neutral | Disagree | Strongly disagree |
| **6.** **You pay more attention to your doctor's advice and are willing to attend regular follow-ups and take medication as prescribed.** | Strongly agree | Agree | Neutral | Disagree | Strongly disagree |
| **7.** **You recognize the role of psychological counseling in cardiac rehabilitation.** | Strongly agree | Agree | Neutral | Disagree | Strongly disagree |
| **8.** **You recognize that avoiding HF triggers (e.g., unhealthy habits, intense physical activity) is an important part of cardiac rehabilitation.** | Strongly agree | Agree | Neutral | Disagree | Strongly disagree |

**Part 4 Practices**

| **1. Frequency with which you proactively acquire knowledge about cardiac rehabilitation through various channels (e.g., attending in-hospital education, watching TV or online resources, consulting your doctor):** | Always | Often | Sometimes | Rarely | Never |
| --- | --- | --- | --- | --- | --- |
| **2. Frequency of monitoring yourself for potential symptoms of HF:** | Always | Often | Sometimes | Rarely | Never |
| **3. Frequency of regular follow-up visits to the hospital for HF:** | Always | Often | Sometimes | Rarely | Never |
| **4. Frequency of maintaining a healthy diet (e.g., low-salt, low-fat):** | Always | Often | Sometimes | Rarely | Never |
| **5. Frequency of engaging in regular exercise:** | Always | Often | Sometimes | Rarely | Never |
| **6. Degree to which you actively manage HF through lifestyle changes such as reducing late nights, smoking, and drinking.** | Always | Often | Sometimes | Rarely | Never |
| **7. Frequency with which you seek psychological counseling for negative emotions associated with HF:** | Always | Often | Sometimes | Rarely | Never |
| **8. Degree to which you comply with doctor’s orders regarding medication schedules and dosages.** | Always | Often | Sometimes | Rarely | Never |
