# Supplementary material for: Knowledge, attitudes and practices towards cardiac rehabilitation among patients with heart failure
Source: BMC Public Health. 2026 May 29;26:2779. doi: 10.1186/s12889-026-27912-1 (PMC13637361; doi:10.1186/s12889-026-27912-1)
Supplement: Supplementary file 2 [file 12889_2026_27912_MOESM2_ESM.docx]

**Supplementary Table 1. Distribution of knowledge dimension responses.**

| **Knowledge** | **N (%)** | | |
| --- | --- | --- | --- |
|  | **True** | **False** | **Unclear** |
| **1.** **Heart failure is the severe and terminal stage of all cardiovascular diseases.** | 350 (59.42) | 58 (9.85) | 181 (30.73) |
| **2.** **High incidence, high hospitalization rate, and high mortality rate are the main Epidemiological features of** **heart failure.** | 372 (63.16) | 65 (11.04) | 152 (25.81) |
| **3.** **When experiencing symptoms like chest tightness, shortness of breath, and fatigue, one should be alert to the occurrence of heart failure.** | 414 (70.29) | 55 (9.34) | 120 (20.37) |
| **4.** **Symptoms in Patients with heart failure are always obvious.** | 350 (59.42) | 239 (40.58) | / |
| **5.** **There are many aspects of cardiac rehabilitation, including exercise training, lifestyle improvement, and psychological counseling, etc.** | 429 (72.84) | 67 (11.38) | 93 (15.79) |
| **6.** **Exercise rehabilitation is an important method of cardiac rehabilitation for patients with heart failure.** | 361 (61.29) | 101 (17.15) | 127 (21.56) |
| **7.** **Patients with malignant arrhythmias or acute heart failure can still participate in exercise rehabilitation.** | 258 (43.8) | 186 (31.58) | 145 (24.62) |
| **8.** **Common types of exercise rehabilitation include aerobic training (e.g., jogging), resistance training (e.g., shoulder press), and flexibility training.** | 374 (63.5) | 67 (11.38) | 148 (25.13) |
| **9.** **Cardiac rehabilitation generally requires regular assessment and timely adjustments based on actual conditions.** | 444 (75.38) | 54 (9.17) | 91 (15.45) |
| **10.** **Cardiac rehabilitation in heart failure patients should pay close attention to the indications and adverse reactions of drug therapy.** | 419 (71.14) | 56 (9.51) | 114 (19.35) |
| **11.** **Cardiac rehabilitation for Patients with heart failure can be solely managed by hospitals and doctors, with no need for the patient to engage much in self-management.** | 263 (44.65) | 227 (38.54) | 99(16.81) |

**Supplementary Table 2. Distribution of attitude dimension responses.**

| **Attitude** | **Strongly agree** | **Agree** | **Neutral** | **Disagree** | **Strongly disagree** |
| --- | --- | --- | --- | --- | --- |
| **1.** **You believe that it is important for patients to pay attention to cardiac rehabilitation and to learn relevant knowledge.** | 237 (40.24) | 227 (38.54) | 74 (12.56) | 24 (4.07) | 27 (4.58) |
| **2.** **You believe that cardiac rehabilitation is mainly handled by doctors, and patients generally do not need to pay much attention.** | 127 (21.56) | 124 (21.05) | 85 (14.43) | 186 (31.58) | 67 (11.38) |
| **3.** **You think that cardiac rehabilitation will require a lot of effort, and you lack confidence in strictly adhering to it.** | 125 (21.22) | 133 (22.58) | 116 (19.69) | 162 (27.5) | 53 (9) |
| **4.** **You are willing to engage in cardiac rehabilitation through appropriate physical activities (e.g., jogging, weight-lifting, or flexibility exercises).** | 165 (28.01) | 262 (44.48) | 107 (18.17) | 45 (7.64) | 10 (1.7) |
| **5.** **You pay significant attention to weight management in your daily life.** | 187 (31.75) | 240 (40.75) | 114 (19.35) | 31 (5.26) | 17 (2.89) |
| **6.** **You pay more attention to your doctor's advice and are willing to attend regular follow-ups and take medication as prescribed.** | 193 (32.77) | 260 (44.14) | 88 (14.94) | 25 (4.24) | 23 (3.9) |
| **7.** **You recognize the role of psychological counseling in cardiac rehabilitation.** | 161 (27.33) | 262 (44.48) | 116 (19.69) | 24 (4.07) | 26 (4.41) |
| **8.** **You recognize that avoiding HF triggers (e.g., unhealthy habits, intense physical activity) is an important part of cardiac rehabilitation.** | 178 (30.22) | 271 (46.01) | 84 (14.26) | 35 (5.94) | 21 (3.57) |

**Supplementary Table 3. Distribution of practice dimension responses.**

| **Practice** | **Always/** **Absolutely Matched** | **Often/** **Matched** | **Sometimes/ Not necessarily matched** | **Occasionally/ Not Matched** | **Never/ Completely Not Matched** |
| --- | --- | --- | --- | --- | --- |
| **1. Frequency with which you proactively acquire knowledge about cardiac rehabilitation through various channels (e.g., attending in-hospital education, watching TV or online resources, consulting your doctor):** | 140(23.77) | 163(27.67) | 125(21.22) | 105(17.83) | 56(9.51) |
| **2. Frequency of monitoring yourself for potential symptoms of HF:** | 148(25.13) | 151(25.64) | 104(17.66) | 94(15.96) | 92(15.62) |
| **3. Frequency of regular follow-up visits to the hospital for HF:** | 148(25.13) | 151(25.64) | 104(17.66) | 94(15.96) | 92(15.62) |
| **4. Frequency of maintaining a healthy diet (e.g., low-salt, low-fat):** | 131(22.24) | 162(27.5) | 104(17.66) | 94(15.96) | 98(16.64) |
| **5. Frequency of engaging in regular exercise:** | 157(26.66) | 195(33.11) | 129(21.9) | 68(11.54) | 40(6.79) |
| **6. Degree to which you actively manage HF through lifestyle changes such as reducing late nights, smoking, and drinking.** | 145(24.62) | 177(30.05) | 127(21.56) | 92(15.62) | 48(8.15) |
| **7. Frequency with which you seek psychological counseling for negative emotions associated with HF:** | 173(29.37) | 219(37.18) | 127(21.56) | 36(6.11) | 34(5.77) |
| **8. Degree to which you comply with doctor’s orders regarding medication schedules and dosages.** | 123(20.88) | 172(29.2) | 94(15.96) | 100(16.98) | 100(16.98) |

**Supplementary table 4. SEM fit indices.**

| Indicators | Reference | Results |
| --- | --- | --- |
| RMSEA | < 0.08 Good | 0.060 |
| SRMR | < 0.08 Good | 0.093 |
| TLI | > 0.8 Good | 0.870 |
| CFI | > 0.8 Good | 0.886 |
